# High prevalence of olfactory impairment among leprosy patients: A cross-sectional study

**DOI:** 10.1371/journal.pntd.0010888

**Published:** 2023-04-05

**Authors:** Rogério Nabor Kondo, Milene Cripa Pizatto de Araújo, Paulo Muller Ramos, Hélio Amante Miot, Marco Aurélio Fornazieri

**Affiliations:** 1 Clinical Medicine Department, Dermatology, Universidade Estadual de Londrina, Londrina, Paraná, Brazil; 2 Dermatology Department of São Paulo State University, Botucatu, São Paulo, Brazil; 3 Department of Clinical Surgery, Universidade Estadual de Londrina, Londrina, Paraná, Brazil; 4 Department of Medicine, Pontifícia Universidade Católica do Paraná, Londrina, Paraná, Brazil; 5 Department of Otorhinolaryngolgy, Universidade de São Paulo, São Paulo, Brazil; 6 Smell and Test Center, Department of Otorhinolaryngology–Head and Neck Surgery, Perelman School of Medicine, University of Pennsylvania; Johns Hopkins University, UNITED STATES

## Abstract

**Background:**

The effect of leprosy on the sense of smell is not yet fully established. Studies that have relied only on patients’ perceptions may have under- or over-estimated the change in smell perception. A validated and psychophysical method is necessary to avoid these errors in assessment.

**Objectives:**

This study aimed to validate the existence of olfactory involvement in leprosy patients.

**Methods:**

A cross-sectional, controlled study was conducted, in which individuals with leprosy (exposed individuals) and individuals without leprosy (control patients) were recruited. For each exposed individual, we selected two control patients. A total of 108 patients (72 control patients and 36 exposed individuals) with no history of infection with the new coronavirus (COVID-19) took the University of Pennsylvania Smell Identification Test (UPSIT).

**Results:**

Most exposed individuals had olfactory dysfunction [*n* = 33, 91.7% (CI 95%: 77.5%–98.3%)] when compared with the control patients [n = 28, 38.9% (CI 95%: 27.6%-51.1%)], but only two (5.6%) had olfactory complaints. The olfactory function was significantly worse among exposed individuals [UPSIT leprosy = 25.2 (CI 95%: 23.1–27.3) when compared with the UPSIT control patients = 34.1 (CI 95%: 33.0–35.3); p<0.001]. The risk of olfactory loss was higher among the exposed individuals [OR: 19.5 (CI 95%: 5.18–105.70; *p* < 0.001)].

**Conclusions:**

Olfactory dysfunction was highly prevalent among exposed individuals, although they had little or no self-knowledge of the disorder. The results show that it is important to assess the sense of smell in exposed individuals.

## Author summary

Leprosy is one of the oldest neurocutaneous diseases in the world. However, it has been neglected by the general population, doctors, and scientists. Transmitted by *Mycobacterium leprae* and *Mycobacterium lepromatosis*, leprosy is met with stigma and prejudice. Some patients with leprosy (exposed individuals) keep their diagnosis a secret, fearing a negative impact on their social lives. The disease is characterized by white or red skin lesions associated with local numbness. Few studies have evaluated smell among exposed individuals, but they have not used the University of Pennsylvania Smell Identification Test (UPSIT), which is a validated psychophysical method. The present study found a high prevalence of olfactory impairment (91.7%) among exposed individuals, accompanied by low self-knowledge of the disorder (5.6%). It is thus important to assess the sense of smell in infected individuals, as this sensorineural assessment has not been performed as a routine for patients.

## Introduction

Leprosy, or Hansen’s Disease (HD), is a chronic granulomatous neurocutaneous disease that is caused by non-culturable acid-alcohol-resistant bacilli, *Mycobacterium leprae* and *Mycobacterium lepromatosis* [1,2]. These infectious agents multiply slowly, and the incubation period is long (about five years, but can exceed 40 years) [3]. Traditionally one of the most stigmatized diseases, leprosy continues to be associated with the ideas of sin, impurity, and punishment [4]. Even though the disease has a cure, some patients keep their diagnosis a secret, fearing a negative and stigmatizing image, as seen throughout history [5].

Despite efforts to eradicate it world over [6], leprosy is a neglected disease. Brazil, where this study was conducted, has the second largest number of cases in the world, preceded by India [7]. At the beginning of the 2000s, approximately 94% of new cases diagnosed in the Americas are reported in Brazil. Northeast, North, and Midwest Brazil report the highest incidence. Fortunately, over the last few decades, prevalence rates have fallen as a result of the consolidation of multidrug therapies (MDT) [8].

Leprosy affects the cooler regions of the body, mainly the skin, peripheral nerves, eyes, and the mucous membrane of the upper respiratory tract [1]. It affects hearing [9], vision [10], taste [11], and smell [12]. The data, based on the evaluation method, suggest that olfactory dysfunction occurs in 40–100% of leprosy cases, which may help diagnose the disease, and facilitate the implementation of timely interventions [12,13]. However, less accurate olfactory tests were used in these studies. Some studies have suggested that patients are often unaware of an olfactory loss [14], which brings into question whether the prevalence of olfactory loss in leprosy is likely to be much higher than expected.

Just as the nasal deformity alters the quality of life of several patients [15], olfactory alterations can have negative consequences for these individuals, especially once it influences their nutrition, safety, and well-being [16]. This study explores the olfactory dysfunctions and encourages an evaluation routine among leprosy patients through a complete, validated olfactory test [17].

## Methods

A cross-sectional, controlled study was conducted, in which individuals with leprosy (exposed individuals) and individuals without leprosy (control patients) were recruited. For each exposed individual (new cases sampled at the time of diagnosis and existing cases undergoing MDT), we selected two control patients. The study was conducted through non-probabilistic convenience sampling, which means that all cases of leprosy in the city of Londrina-PR, from March 1, 2021 to February 28, 2022, were included in the study, provided that they met the inclusion/exclusion criteria and that the patients involved agreed to participate. A signed consent form was obtained from all patients before participation. The study was approved by the Ethics Committee in Research Involving Human Beings at State University of Londrina, CAAE: 42159420.0.0000.523.

We excluded patients aged under 18 years [17], those who had olfactory disease caused by traumatic brain injury, radiation therapy to the face, and post-airway infection dysfunctions (including COVID-19) [18], and those who did not understand the procedures involved in the test (16 individuals in all).

For data collection, patients were asked to fill out a form and medical information was retrieved from their electronic medical records. A test with 40 odors, namely the University of Pennsylvania Smell Identification Test (UPSIT) [17], was conducted to assess the olfactory function. Each participant was given 4 booklets with 10 substances, which they had to scrape and sniff, before identifying them by marking an option from among four alternatives. They had to choose one (see supplementary material). The UPSIT value ranged from 0 to 40, based on the number of correct answers, classifying the olfactory function of the individual as normosmia, hyposmia, anosmia, or possible simulator. This categorization differed when applied to men and women, wherein the latter presented greater olfactory acuity [17].

We performed Fisher’s Exact Test to compare categorical variables. Continuous variables were analyzed using the Wilcoxon-Mann-Whitney Test. After verifying the normality of the quantitative variables using the Shapiro-Wilk test, the nonparametric Mann-Whitney and Kruskal-Wallis tests were used to compare the results obtained. Spearman’s nonparametric correlation test was used to verify the relationship between discrete quantitative variables. A comparison of the risk of olfactory loss between the exposed and control groups was carried out using logistic regression. We considered the values ​​of statistical significance (*p*-value) under 0.05. The 95% confidence interval was calculated. All data were compiled in an Excel spreadsheet for further statistical analysis. Stata (version 13.0, Statacorp Texas), Jamovi, and R studio were used for statistical analysis.

## Results

We recruited 108 individuals (36 exposed individuals, 72 control patients). Table 1 presents the main data. The average age of the individuals was 53.3 years (range: 24–91 years). Further, 52.8% were men and 47.2% were women. Most exposed individuals had olfactory disorders [*n* = 33 or 91.7% (CI 95%: 77.5–98.3)] when compared to the control group [n = 28 or 38.9% (CI 95%: 27.6%-51.1%)], but only two (5.6%) had olfactory complaints. Multibacillary (86.1%) prevailed, with the borderline-borderline form being the most prevalent in the clinical classification, with 20 cases (55.6%).

**Table 1:** Clinical and demographic characteristics (N = 108).

Characteristics	Leprosy (n = 36)	Control (n = 72)
Age, years		
mean±SD	53.3±18.1	53.3±18.1
minimum–maximum	24–91	24–91
Sex, n° (%)		
male	19 (52.8)	38 (52.8)
female	17 (47.2)	34 (47.2)
Race, n° (%) #		
white	21 (58.6)	43 (59.7)
non-white	15 (41.4)	29 (40.3)
Salary income per family, n° (%)$		
2 or less	23 (63.9)	32 (44.4)
3–4	8 (22.2)	38 (52.8)
5–9	3 (8.3)	2 (2.8)
10–19	1 (2.8)	0 (0.0)
More than 20	1 (2.8)	0 (0.0)
Level of schooling, n° (%)		
incomplete middle school	13 (36.1)	13 (18.1)
< middle school	6 (16.7)	5 (6.9)
some high school	3 (8.3)	1 (1.4)
complete high school	7 (19.4)	23 (32.0)
some college	1 (2.8)	6 (8.3)
bachelors or higher	6 (16.7)	24 (33.3)
Leprosy classification, n° (%) §		
multibacillary	31 (86.1)	not applicable
paucibacillary	5 (13.9)	not applicable
Grade 1 disability, n° (%) &		
with incapacity	6 (16.7)	not applicable
Olfactory complaints, n° (%) *		
with complaints	2 (5.6)	not applicable
Smoking history, n° (%) with history UPSIT	4 (11.1)	26 (36.1)
altered,n° (%)	33 (91.7)	28 (38.9)
mean±SD	25.2±6,2	34,1±1.2
minimum–maximum	8–36	21–40
Nasal deformity, n° (%)		
with deformity ¶	3 (8.3)	not applicable
Laboratory tests, n° (%)		
altered†	6 (16.7)	not applicable

# self-defined race by participants.

$ minimum wage in Brazil in 2021.

§ Borderline-borderline (20/36); Lepromatous leprosy (4/36); Borderline-lepromatous (4/36); Tuberculoid (4/36); Borderline-tuberculoid (3/36); Indeterminate (1/36).

* olfactory complaint self-reported by the participant before the test was conducted.

¶ deviated septum (2/36); nasal polyp (1/36)

† changes related to cholesterol, blood glucose, thyroid-stimulating hormone (TSH), and iron deficiency anemia.

The two exposed individuals (5.6%) who were conscious of their olfactory loss were both men and had the borderline form. One was 45 years of age and a smoker of 14 pack-years, and had severe hyposmia (UPSIT = 22). The other was 62 years of age, and had anosmia (UPSIT = 8).

Olfactory function was significantly worse among exposed individuals [UPSIT leprosy = 25.2 (CI 95%: 23.1–27.3) vs. UPSIT controls = 34.1 (CI 95%: 33.0–35.3); *p* < 0.001]. The risk of olfactory loss was higher among the exposed individuals [odds ratio (OR): 19.5 (CI 95%: 5.18–105.70; *p* < 0.001)]. Olfactory function was negatively and positively correlated with age (*p* = 0.032) and educational level (*p* = 0.028), respectively. Most exposed individuals had anosmia or severe hyposmia (5 and 14, respectively), whereas control patients had normosmia or mild hyposmia (44 and 20, respectively; see Tables 1,2 and 3).

**Table 2:** UPSIT based on sex distribution (N = 108).

UPSIT	Leprosy group		Control group	
	Female n = 17	Male n = 19	Female n = 34	Male n = 38
Anosmia (n, %)	2 (1.8)	3 (2.8)	0 (0.0)	0 (0.0)
Severe hyposmia (n, %)	5 (4.6)	9 (8.3)	2 (1,8)	3 (2.8)
Moderate hyposmia (n, %)	6 (5.6)	2 (1.8)	0 (0.0)	3 (2.8)
Mild hyposmia (n, %)	2 (1.8)	4 (3.6)	7 (6.5)	13 (12.0)
Normosmia (n, %)	2 (1.8)	1 (0.9)	26 (24.1)	18 (16.7)

**Table 3:** UPSIT correlation coefficients with select characteristics (N = 108).

Variables	Leprosy group (n = 36)		Control group (n = 72)	
	Rho	p	Rho	p
Age	-0.36	0.032	-0.56	<0.001
Level of schooling	0.37	0.028	0.36	0.002
Family income	0.06	0.720	0.14	0.222
Treatment duration	0.01	0.965	Na	Na

UPSIT: *University of Pennsylvania Smell Identification Test*, Rho: Spearman´s rho correlation coefficient, Na: not applicable.

Schooling and income variables were categorized from 1 to 6, and 1 to 5, respectively.

**Fig 1:**
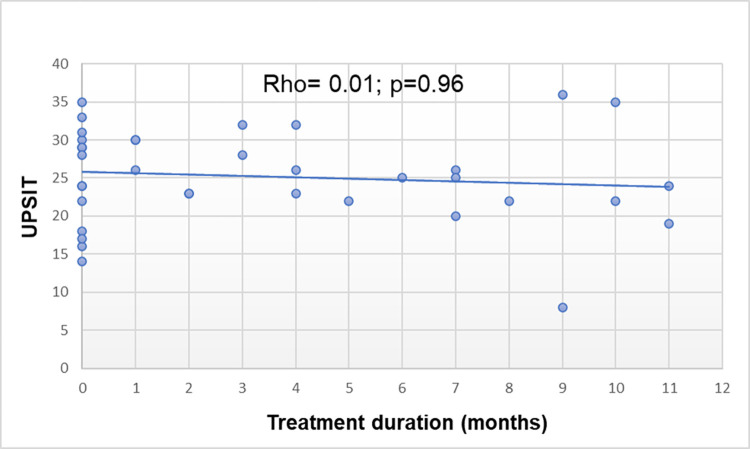
Correlation between UPSIT and treatment duration in months. Rho: Spearman´s correlation coefficient.

**Fig 2:**
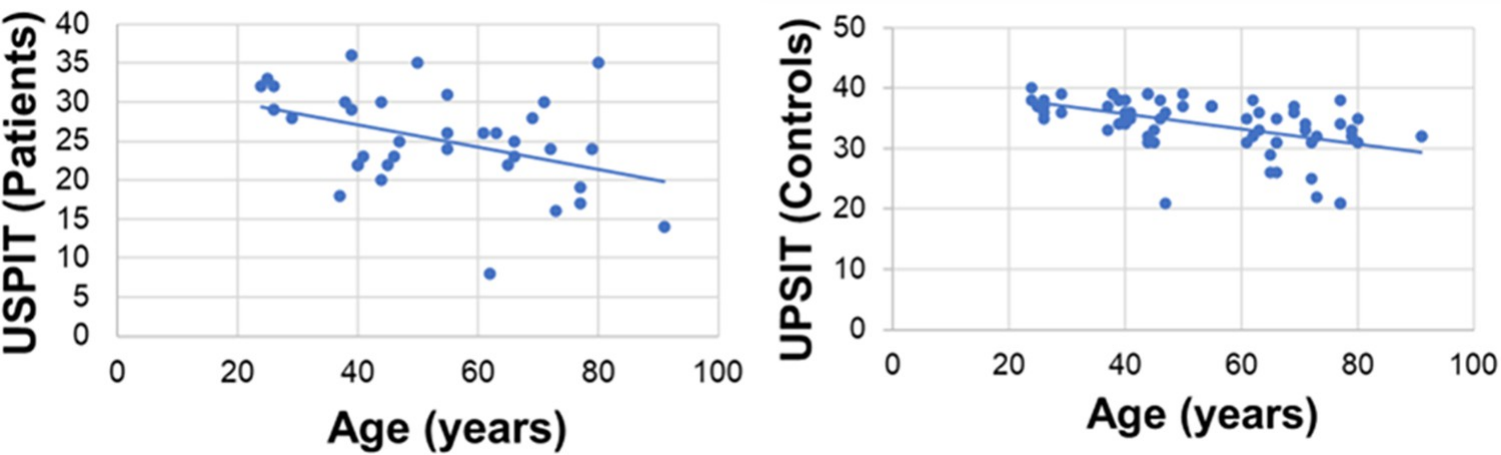
Scatter plot showing UPSIT based on age between the exposed individuals and control patients. **Both groups show a negative correlation (Rho = -0.36, p = 0.032 and Rho = -0.56, p<0.001, respectively).** Rho: Spearman´s correlation coefficient.

Correlation analysis was conducted to verify the correlation between age and UPSIT scores (Spearman’s rho = -0.36; CI 95%: -0.521 to -0.031; *p* = 0.032). In relation to family income and olfactory function, correlation analysis was performed (Spearman’s rho = 0.06; *p* = 0.720; see Table 3). No correlation was found between treatment duration and UPSIT scores (Spearman’s rho = 0.01; *p* = 0.965, Table 3, Figs 1 and 2).

Women scored 1.97 and 1.68 more points in the UPSIT than did men among the exposed individuals (Spearman’s rho = 0.028; *p* = 0.46) and control patients (Spearman’s rho = 0.196; *p* = 0.09), respectively. To analyze the response variable (UPSIT) as a function of the ordinal qualitative explanatory variables (education), correlation analysis was performed [exposed individuals (Spearman = 0.37; CI 95%: 0.048–0.561; *p* = 0.028)] vs. [control patients (Spearman’s rho = 0.36; CI 95%: 0.326–1.500; *p* = 0.002)] (see Table 3).

We performed a normality test (Shapiro-Wilk) among exposed individuals to verify the response variable (UPSIT) as a function of the explanatory multivariable of sex, age, and schooling (nominal, discrete, and ordinal qualitative, respectively). The residuals followed normal distribution, with adjusted R^2^ (0.639) and Akaike information criterion (184). The best female UPSIT interaction model was 29.7 + (2.3 x sex) + (-0.12 x age) + (1.04 x education) and *p* = 0.033.

Among the exposed individuals, only six patients (16.7%) had a degree of disability, namely grade 1. Four patients (11.1%) had a history of smoking, with 10, 11, 14, and 24 cigarette pack-years. None of the patients had septum perforation and nasal collapse. Deformities (8.3%) were related to septal deviation and the presence of polyps (see Table 1).

To evaluate UPSIT as a function of other variables (income, degree of disability, smoking, olfactory complaints, reactions, deformities, and duration of treatment and the disease), other multivariate analyses were performed but had no statistical significance (ps> 0.05). Table 4 shows that gasoline (25% correct) and leather (25% correct), and pickles (54.2% correct) and motor oil (56.9% correct) were the substances with the greatest errors in identification among exposed individuals and control patients, respectively.

**Table 4:** Percentage of correct answers for each odorant among exposed individuals and control patients (N = 108).

Odorants	Leprosy group (n = 36)	Control group (n = 72)	p*
01.Pizza	33.3	70.8	<0.001
02.Bubble gum	55.6	84.7	0.002
03.Menthol	63.9	94.4	<0.001
04.Cherry	72.2	94.4	0.002
05.Motor oil	69.4	56.9	0.295
06.Mint	83.3	97.2	0.016
07.Banana	61.1	86.1	0.006
08.Clove	63.9	95.8	<0.001
09.Leather	25.0	93.1	<0.001
10.Coconut	52.8	93.1	<0.001
11.Onion	83.3	98.6	0.005
12.Fruit juice	58.3	88.9	<0.001
13.Baby powder	86.1	97.2	0.040
14.Jasmine	52.8	88.9	<0.001
15.Cinnamon	75.0	87.5	0.110
16.Gasoline	25.0	90.3	<0.001
17.Strawberry	75.0	83.3	0.314
18.Coffee	38.9	87.5	<0.001
19.Gingerbread	72.2	80.6	0.337
20.Apple	72.2	65.3	0.519
21.Perfume	80.6	90.3	0.223
22.Flower	61.1	93.1	<0.001
23.Peach	75.0	84.7	0.293
24.Tire rubber	80.6	95.8	0.015
25.Pickles	80.6	54.2	0.011
26.Pineapple	66.7	98.6	<0.001
27.Raspberry	86.1	86.1	1.00
28.Orange	30.6	84.7	<0.001
29.Walnut	52.8	65.3	0.218
30.Watermelon	66.7	86.1	0.024
31.Solvent	66.7	73.6	0.502
32.Grass	66.7	56.9	0.406
33.Smoke	77.8	88.9	0.154
34.Wood	61.1	90.3	0.001
35.Grape	66.7	90.3	0.006
36.Garlic	86.1	88.9	0.757
37.Soap	83.3	90.3	0.352
38.Natural gas	75.0	90.3	0.046
39.Rose	63.9	79.2	0.106
40.Peanut	75.0	91.7	0.035

*Statistical significance accessed with Fisher exact test.

## Discussion

Olfactory dysfunction was highly prevalent among exposed individuals (91.7%), although they had little or no self-knowledge of the disorder (5.6%). Olfactory function was significantly worse among exposed individuals [UPSIT leprosy = 25.2 (CI 95%: 23.1–27.3)] when compared with the control patients [UPSIT control patients = 34.1 (CI 95%: 33.0–35.3); p<0.001]. The risk of olfactory loss (Odds ratio or OR) was higher among exposed individuals [OR: 19.5 (CI 95%: 5.18–105.70; p < 0.001)].

Mishra et al. (2006) [14] investigated olfactory dysfunction in leprosy patients, and reported olfactory impairment in all exposed individuals in their sample. They used an olfactory test involving 12 substances (the Brief-Smell Identification Test, or B-SIT). Unfortunately, this smaller test does not classify an individual as having anosmia, or mild, moderate, or severe hyposmia, as in the UPSIT [19] (see Supplementary Material).

As identified by previous studies that observed the olfactory alteration in leprosy [13,14], we need to analyze and understand the prevalence of olfactory alteration in leprosy. Acknowledging this, we realized that olfactory assessments are not routinely undertaken, as is this case, for example, concerning the ophthalmic part, also affected by leprosy. Thus, the study shows the importance of conducting chemosensory tests on a regular basis.

In exposed individuals, there was a negative between the UPSIT and age (Spearman’s rho = -0.36; CI 95%: -0.521 to -0.031; *p* = 0.032). Doty and Kamath (2014) described a decrease in smell with age in the general population [20]. Therefore, the tendency to lose the sense of smell with advancing age may exist among exposed individuals (Table 3).

Our study showed that women tended to have better UPSIT scores [17]. Women scored 1.97 more point than men. Although previous research showed an association [21], the family income of this sample studied was not relevant vis-a-vis the UPSIT (Spearman = 0.06; *p* = 0.46) (Table 3). The education attainment of the individuals in the sample studied showed a positive correlation with the UPSIT, which was statistically significant (Spearman = 0.37; CI 95%: 0.048–0.561; *p* = 0.028). Fornazieri et al. (2019) claimed that a better olfactory function exists among individuals in the general population with a higher level of educational attainment because these individuals may use cognitive strategies and life experiences to differentiate the sense of smell. This pattern of better UPSIT values with higher levels of education may exist among exposed individuals [21] (Table 3).

Treatment duration did not have a significant positive correlation with the UPSIT (Spearman = 0.008; *p* = 0.965; see Table 3 and Fig 1). When Mishra et al. (2006) applied the B-SIT test, they did not show any improvement in olfactory function with treatment [14]. For the prognosis of improvement, this finding is problematic, given that the treatment may not improve this function and that sometimes, it does not improve the thermal, painful, and tactile sensitivities, because of the destruction of the sensory nerves. Mishra et al. showed greater loss in the Virchowian pole [14]. However, our results indicated no correlation with the UPSIT (Spearman = 0.178; *p* = 0.070) because 55% of the individuals presented borderline-borderline, and only 11% presented the lepromatous leprosy.

The anatomical alterations were low among exposed individuals (*n* = 3, or 8.3%). There were no cases of septal perforation and nasal alae collapse. The history of smoking in the sample was low (*n* = 4, or 11.1%). Only six patients (16%) had a degree of disability (grade 1). Owing to these low numbers, it was not possible to establish a correlation between these findings and the UPSIT (see Table 1).

The authors are concerned that gasoline was one of the substances with the greatest errors in identification (25% accuracy), as it is a flammable, combustible material. A patient with leprosy may have a certain degree of visual and tactile incapacity and, without realizing it, may put their life at risk.

Multiple factors may be involved in anosmia (total loss of smell) or hyposmia (decreased smell) in leprosy, such as the direct action of the agent causing damage to the olfactory receptors and bulb in the disease process. Bipolar olfactory cells are not myelinated and therefore germinate from basal cells and project directly into the olfactory bulb through the cribriform plate, as these are invasion routes for toxic agents, viruses, and bacilli (such as *M*. *leprae* and *M*. *lepromatosis*).

Leprosy is a secondary cause of atrophic rhinitis [14]. The main characteristics of leprosy rhinitis are chronicity, the absence of acute phenomena, and the incomplete obstruction of the nasal mucosa, presenting dryness and crust-formation [22]. As the condition progresses, it frequently produces perforations of the septum, and causes the destruction of the cartilaginous part and collapse of the nasal ala [23].

We hypothesize that olfactory loss in patients with leprosy may occur because of the involvement and destruction of receptor axons and the olfactory bulb by the bacillus, as they are situated in the paths taken by the agent, as is the case with other aggressive agents.

Olfactory dysfunction may be related to the lack of full recovery of smell because of the latency of the onset of symptoms and their treatment, as it happens in some infections like COVID-19 [18,24]. As leprosy is a neglected disease, there may be a delay in the diagnosis and initiation of treatment; and a delay of six months of typical nervous symptoms of leprosy is usually irreversible [1]. The lack of correlation between the duration of the disease and a poor UPSIT score in the present study may have been related to the patients’ delay in noticing the initial spots and suspecting leprosy only when there was a lack of sensitivity. This may mean that the duration of the disease reported by the patients may have been underestimated in relation to the point in time when it was contracted.

The present study did not observe septum perforations and nasal ala collapse, both of which can negatively influence olfactory function. We encourage a frequent clinical evaluation of patients with leprosy through the UPSIT, because most exposed individuals presented high prevalence of the disease (91.7%), whereas only 5.6% were able to acknowledge the loss of the sense of smell. Leprosy is thus a silent and reckless disorder, as smell is also a sense of alertness and protection.

One limitation of this study is that it was cross-sectional, and used a convenience sample based only on participants living in Londrina-PR. The exclusion of participants also limited our study, as we excluded symptomatic participants infected by COVID-19 (*n* = 14) in addition to one individual who had traumatic brain injury (stroke) and was unable to understand the complexity of the test (*n* = 1). Previous infection with the SARS-CoV-2 can permanently impair olfactory capacity. Confirmation bias may have occurred in the study owing to the different cognitive levels among the participants.

## Conclusion

Most individuals examined presented olfactory dysfunction (91.7%). Only 5.6% were able to acknowledge the loss of the sense of smell, suggesting that it is important to assess the sense of smell in patients with leprosy. Olfactory alterations were correlated with patients with lower educational attainment and older age groups.

## Supporting information

S1 Fig UPSIT offers 4 booklets, containing 10 pages each, totaling 40 substances in all. **Source:** Author’s own illustration based on current booklets.
(TIFF)

S2 Fig To release the odorant, the patient scrapes the square in the lower corner of each page using a pencil suitable for the test. Once the odorant is released, the patient must choose one of the four alternatives presented to identify the odor. **Source:** Author’s own illustration based on current booklets.
(TIF)

S3 Fig Researcher holds the booklet as an example of how the test is performed. After scraping the square in the lower page, the individual smells the odorant and tries to identify the substance. Then, they select the appropriate option on the page. **Source:** Author’s own photo file.
(TIFF)

S1 Table UPSIT denotes University of Pennsylvania Smell Identification Test. (DOCX)

S2 Table UPSIT denotes University of Pennsylvania Smell Identification Test. (DOCX)

S1 STROBE STROBE Statement—checklist of items that should be included in reports of observational studies. (PDF)

## References

[1] Pavezzi PD, do Prado RB, Boin Filho PÂ, Gon AS, Tuma B, Fornazieri MA, et al. Evaluation of ocular involvement in patients with Hansen’s disease. PLoS Negl Trop Dis. 2020;14(9):e0008585. doi: 10.1371/journal.pntd.0008585 32956360 PMC7505469

[2] Han XY, Seo YH, Sizer KC, Schoberle T, May GS, Spencer JS, et al. A new mycobacterium species causing diffuse lepromatous leprosy. Am J Clin Pathol. 2008;130: 856–864. doi: 10.1309/AJCPP72FJZZRRVMM 19019760

[3] WHO. Global leprosy update, 2015. Weekly Epidemiologic Rec. 2016;91:405–420.

[4] Foss NT. Hanseníase: aspectos clínicos, imunológicos e terapêuticos. An Bras Dermatol. 1999; 74 (2): 113–9.

[5] Navon L. Beggars, metaphors, and stigma: a missing link in the social history of leprosy. Soc Hist Med.1998;11(1):89–105. doi: 10.1093/shm/11.1.89 11620156

[6] Naaz F, Mohanty PS, Bansal AK, Kumar D, Gupta UD. Challenges beyond elimination in leprosy. Int J Mycobacteriol. 2017;6(3):222–28. doi: 10.4103/ijmy.ijmy_70_17 28776519

[7] Prevedello FC, Mira MT. Hanseníase: uma doença genética? An Bras Dermatol.2007;82(5):451–9.

[8] Araújo MG. Hanseníase no Brasil. Rev Soc Bras Med Trop. 2003;36(3):373–382. 12908039

[9] Koyuncu M, Celik O, Inan E, Ozturk A. Doppler sonography of vertebral arteries and audiovestibular system investigation in leprosy. Int J Lepr Other Mycobact Dis 1995;63: 23–27. 7730715

[10] Yowan P, Danneman K, Koshy S, Richard J, Daniel E. Knowledge and practice of eye-care among leprosy patients. Indian J Lepr 2002;74:129–135. 12708731

[11] Soni NK. Leprosy of the tongue. Indian J Lepr 1992;64: 325–330. 1431321

[12] Veyseller B, Aksoy F, Yildirim YS, Açikalin RM, Gürbüz D, Ozturan O. Olfactory dysfunction and olfactory bulb volume reduction in patients with leprosy Indian J Otolaryngol Head Neck Surg. 2012 Sep;64(3):261–5. doi: 10.1007/s12070-011-0284-9 Epub 2011 Aug 27. 23998032 PMC3431526

[13] Barton RP. Clinical manifestation of leprous rhinitis. Ann Otol Rhinol Laryngol 1976;85:74–82. doi: 10.1177/000348947608500113 1259317

[14] Mishra A, Saito K, Barbash SE, Mishra N, Doty R. Olfactory Dysfunction in Leprosy. Laryngoscope,2006;116:413–416. doi: 10.1097/01.MLG.0000195001.03483.F2 16540900

[15] Bottene IMC, Reis VMS. Quality of life of patients with paucibacillary leprosy. An Bras Dermatol.2012;48(3):408–11. doi: 10.1590/s0365-05962012000300009 22714756

[16] Doty RL. Age-Related Deficits in Taste and Smell. Otolaryngol Clin North Am. 2018 Aug;51(4):815–825. doi: 10.1016/j.otc.2018.03.014 30001793

[17] Fornazieri MA, Doty RL, Santos CA, Pinna FR, Bezerra TFP, Voegels RL. A new cultural adaptation of the University of Pennsylvania Smell Identification Test. Clinics.2013; 68(1):65–8. doi: 10.6061/clinics/2013(01)oa10 23420159 PMC3552441

[18] Boscolo-Rizzo P, Menegaldo A, Fabbris C, Spinato G, Borsetto D, Vaira LA, et al. Six-Month Psychophysical Evaluation of Olfactory Dysfunction in Patients with COVID-19. Chem Senses. 2021 Jan 1;46:bjab006. doi: 10.1093/chemse/bjab006 33575808 PMC7929204

[19] Menon C, Westervelt HJ, Jahn DR, Dressel JA, O’Bryant SE. Normative performance on the Brief Smell Identification Test (BSIT) in a multi-ethnic bilingual cohort: a Project FRONTIER study. Clin Neuropsychol. 2013;27(6):946–961. doi: 10.1080/13854046.2013.796406 23634698 PMC3742676

[20] Doty RL, Kamath V. The influences of age on olfaction: a review. Front. Psychol. 5:20. doi: 10.3389/fpsyg.2014.00020 24570664 PMC3916729

[21] Fornazieri MA, Doty RL, Bezerra TFP, Pina FR, Costa FO, Voegels RL, et al. Relationship of socioeconomic status to olfactory function. Physiol Behav. 2019 Jan 1;198:84–89. doi: 10.1016/j.physbeh.2018.10.011 30336228

[22] Camacho ID, Burdick A, Benjamin L, Casiano R. Chronic rhinitis: a manifestation of leprosy. Ear Nose Throat J.2011;90(9):E1–3. doi: 10.1177/014556131109000915 21938685

[23] Torre J. Manifestaciones nasales de la lepra. Rev Cuba Med Gen Integr. 2015;31(1):52–60.

[24] Horikiri K, Kikuta S, Kanaya K, Shimizu Y, Nishijima H, Yamasoba T, Kondo K. Intravenous olfactory test latency correlates with improvement in post-infectious olfactory dysfunction. Acta Otolaryngol. 2017;137(10):1083–1089. doi: 10.1080/00016489.2017.1325005 28503989

