# Supplementary figures and images for: High prevalence of olfactory impairment among leprosy patients: A cross-sectional study
Source: PLoS Negl Trop Dis. 2023 Apr 5;17(4):e0010888. doi: 10.1371/journal.pntd.0010888 (PMC10075396; doi:10.1371/journal.pntd.0010888)

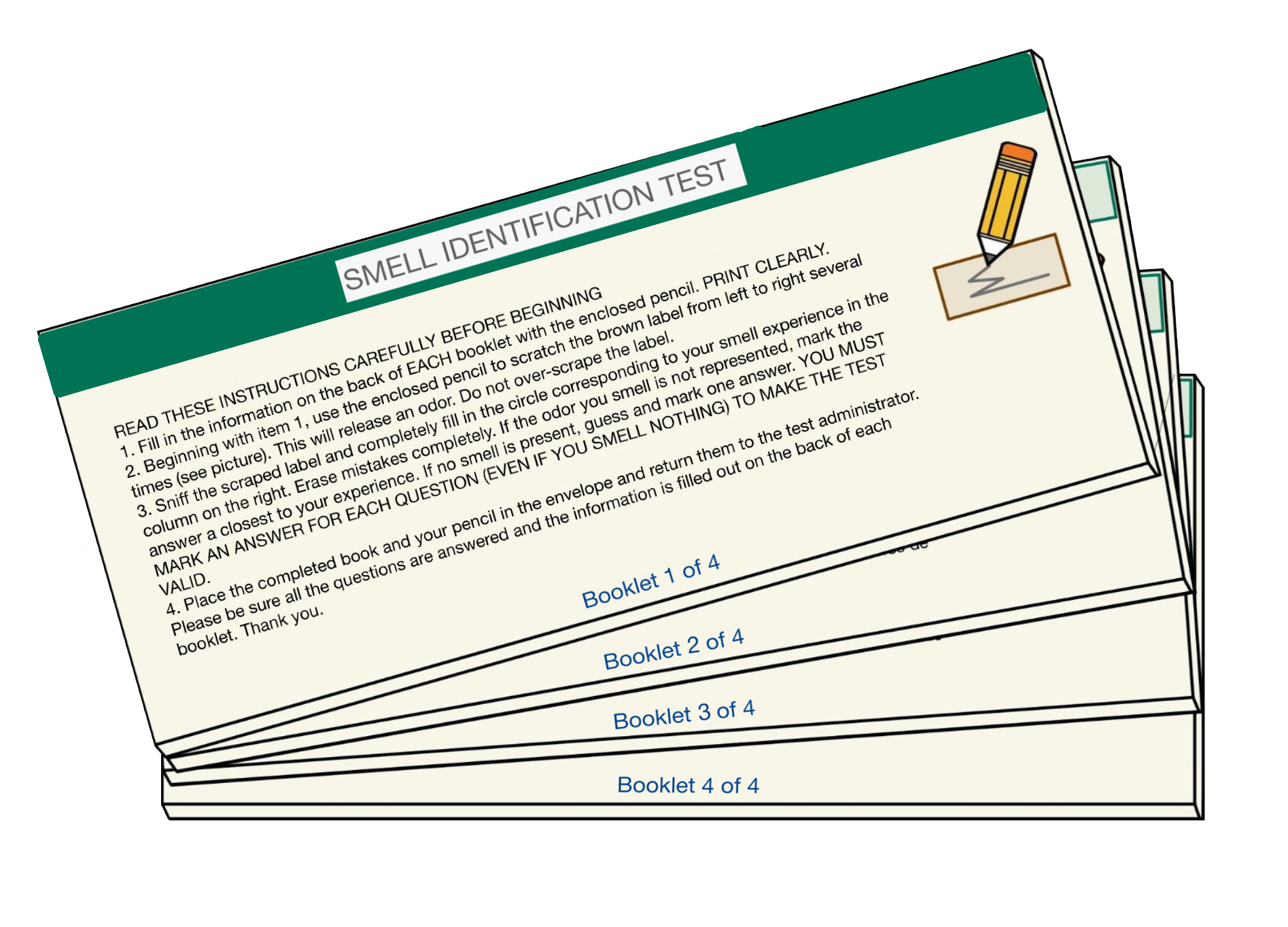

Supplement: S1 Fig — Source: Author’s own illustration based on current booklets. (TIFF) [file pntd.0010888.s001.tiff]

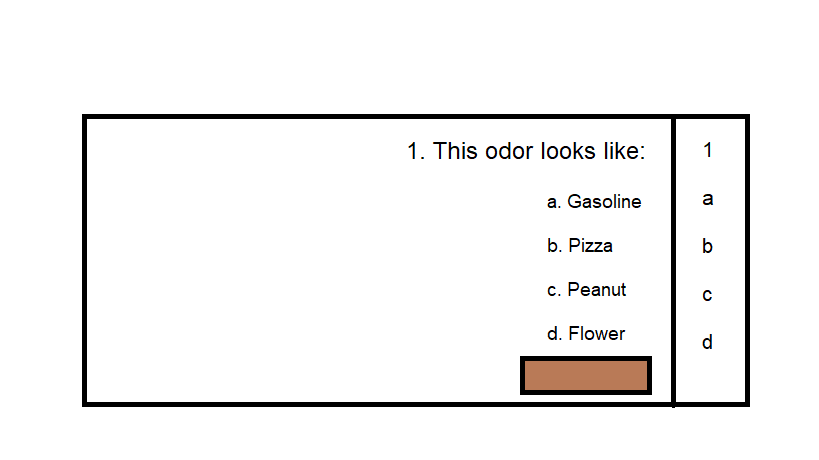

Supplement: S2 Fig — Once the odorant is released, the patient must choose one of the four alternatives presented to identify the odor. Source: Author’s own illustration based on current booklets. (TIF) [file pntd.0010888.s002.tif]

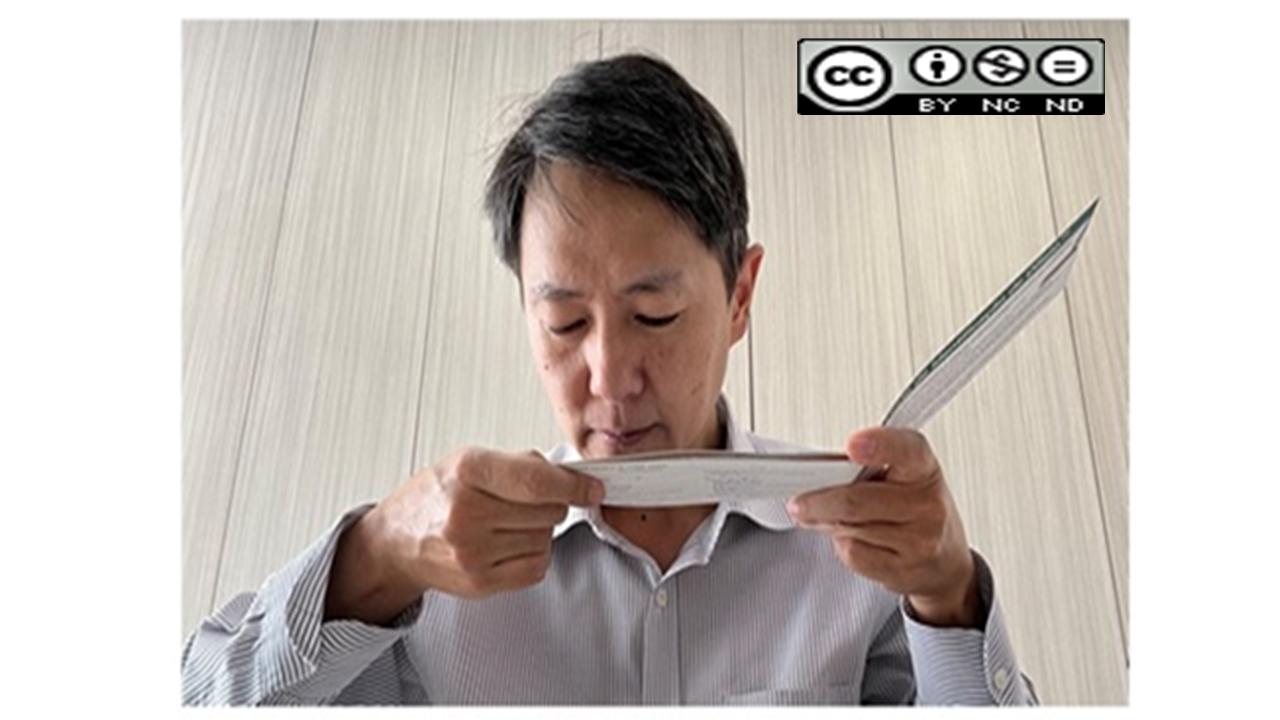

Supplement: S3 Fig — After scraping the square in the lower page, the individual smells the odorant and tries to identify the substance. Then, they select the appropriate option on the page. Source: Author’s own photo file. (TIFF) [file pntd.0010888.s003.Tiff]
