# Supplementary material for: High prevalence of olfactory impairment among leprosy patients: A cross-sectional study
Source: PLoS Negl Trop Dis. 2023 Apr 5;17(4):e0010888. doi: 10.1371/journal.pntd.0010888 (PMC10075396; doi:10.1371/journal.pntd.0010888)
Supplement: S1 Table — (DOCX) [file pntd.0010888.s004.docx]

| **S1Table. Standard substances in the UPSIT** | | | |
| --- | --- | --- | --- |
| **Booklet 1** | **Booklet 2** | **Booklet 3** | **Booklet 4** |
| 1.Pizza | 11.Onion | 21.Perfume | 31.Solvent |
| 2.Bubble gum | 12.Fruit juice | 22.Flower | 32.Grass |
| 3.Menthol | 13.Baby powder | 23.Peach | 33.Smoke |
| 4.Cherry | 14.Jasmine | 24.Tire rubber | 34.Wood |
| 5.Motor oil | 15.Cinnamon | 25.Pickles | 35.Grape |
| 6.Mint | 16.Gasoline | 26.Pineapple | 36.Garlic |
| 7.Banana | 17.Strawberry | 27.Raspberry | 37.Soap |
| 8.Clove | 18.Coffee | 28.Orange | 38.Natural gas |
| 9.Leather | 19.Gingerbread | 29.Walnut | 39.Rose |
| 10.Coconut | 20.Apple | 30.Watermelon | 40.Peanut |
| UPSIT denotes University of Pennsylvania Smell Identification Test | | | |
