# Supplementary material for: High prevalence of olfactory impairment among leprosy patients: A cross-sectional study
Source: PLoS Negl Trop Dis. 2023 Apr 5;17(4):e0010888. doi: 10.1371/journal.pntd.0010888 (PMC10075396; doi:10.1371/journal.pntd.0010888)
Supplement: S2 Table — (DOCX) [file pntd.0010888.s005.docx]

| **S2Table. UPSIT classification for men and women among the Brazilian population** | | |  |
| --- | --- | --- | --- |
| **Classification** | **Total points (Male)** | **Total points (Female)** | |
| Normosmia | 32 to 40 points | 35 to 40 points | |
| Mild hyposmia | 28 to 31 points | 31 to 34 points | |
| Moderate hyposmia | 24 to 27 points | 26 to 30 points | |
| Severe hyposmia | 17 to 23 points | 19 to 25 points | |
| Anosmia | 6 to 16 points | 6 to 18 points | |
| Probable simulator | 0 to 5 points | 0 to 5 points | |
| UPSIT denotes University of Pennsylvania Smell Identification Test | | |  |
